# Correlations of Behavioral Deficits with Brain Pathology Assessed through Longitudinal MRI and Histopathology in the R6/2 Mouse Model of HD

**DOI:** 10.1371/journal.pone.0060012

**Published:** 2013-04-04

**Authors:** Ivan Rattray, Edward Smith, Richard Gale, Kaoru Matsumoto, Gillian P. Bates, Michel Modo

**Affiliations:** 1 King’s College London, Institute of Psychiatry, Department of Neuroscience, London, United Kingdom; 2 King’s College London, Department of Medical and Molecular Genetics, London, United Kingdom; 3 University of Pittsburgh, Department of Radiology, McGowan Institute for Regenerative Medicine, Pittsburgh, Pennsylvania, United States of America; Centre Hospitalier de l’Université Laval, Canada

## Abstract

Huntington’s disease (HD) is caused by the expansion of a CAG repeat in the huntingtin (*HTT*) gene. The R6/2 mouse model of HD expresses a mutant version of exon 1 *HTT* and develops motor and cognitive impairments, a widespread huntingtin (HTT) aggregate pathology and brain atrophy. Despite the vast number of studies that have been performed on this model, the association between the molecular and cellular neuropathology with brain atrophy, and with the development of behavioral phenotypes remains poorly understood. In an attempt to link these factors, we have performed longitudinal assessments of behavior (rotarod, open field, passive avoidance) and of regional brain abnormalities determined through magnetic resonance imaging (MRI) (whole brain, striatum, cortex, hippocampus, corpus callosum), as well as an end-stage histological assessment. Detailed correlative analyses of these three measures were then performed. We found a gender-dependent emergence of motor impairments that was associated with an age-related loss of regional brain volumes. MRI measurements further indicated that there was no striatal atrophy, but rather a lack of striatal growth beyond 8 weeks of age. T2 relaxivity further indicated tissue-level changes within brain regions. Despite these dramatic motor and neuroanatomical abnormalities, R6/2 mice did not exhibit neuronal loss in the striatum or motor cortex, although there was a significant increase in neuronal density due to tissue atrophy. The deposition of the mutant HTT (mHTT) protein, the hallmark of HD molecular pathology, was widely distributed throughout the brain. End-stage histopathological assessments were not found to be as robustly correlated with the longitudinal measures of brain atrophy or motor impairments. In conclusion, modeling pre-manifest and early progression of the disease in more slowly progressing animal models will be key to establishing which changes are causally related.

## Introduction

Huntington’s disease (HD) is a devastating autosomal dominant disorder, caused by a CAG/polyglutamine repeat expansion (HDCRG, 1993). This unstable elongation leads to the aggregation of mutant huntingtin (mHTT), eventually resulting in a substantial neurodegeneration and death. Even in premanifest patients, recently subtle changes in brain structures were revealed to be associated with disease burden [1], [2], [3]. Still, the causal cascade between mHTT and the development of clinical signs remains poorly understood. The variability in disease symptoms and progression, as well as the unavailability of affected tissue during the early stage of disease, impede efforts to link molecular pathology to both brain atrophy and behavioral dysfunction.

To overcome these issues and improve our understanding of disease progression, rodent models of HD have been developed. Transgenic mouse models include the R6/2 [4] and N171Q82 [5] lines that express N-terminal fragments of HTT and the YAC128 [6], and BACHD [7] lines that express a mutant version of the full-length protein. The genetic basis of HD is more precisely recapitulated by the knock-in models in which an expanded CAG repeat has been inserted into mouse *Htt* and that develop a more slowly progressing phenotype [8], [9], [10]. We have found there to be numerous comparable late-stage phenotypes between R6/2 mice at 12–14 weeks and *Hdh*Q150 mice at 22 months of age [11], [12], [13], [14], [15]. Consequently, we defined the N-terminal mHTT fragments that are present in *Hdh*Q150 brain tissue and found that the smallest fragment is exon 1 protein [16]. We have subsequently shown that this is generated through the mis-splicing exon 1, indicating that the R6/2 mice are a model for the aberrant splicing that occurs in HD (unpublished). This current study is part of a continuing detailed comparison of the progressive pathologies exhibited by the R6 and knock-in mouse models.

Due to its rapid and highly reproducible phenotype, the R6/2 line is the most common choice for preclinical HD studies. These mice develop age-related deficits in motor coordination, locomotor activity, anxiolytic behavior and impaired cognition [17], [18], [19], [20]. A progressive regional brain atrophy is evident and can be assessed non-invasively using magnetic resonance imaging (MRI) [21], [22], [23], [24], [25], [26]. Although individual features of the R6/2 mouse model have been extensively characterized, a longitudinal assessment of the emergence of behavioral dysfunction with concomitant measurement of brain atrophy by MRI, along with their correlation with cellular and molecular changes, is essential to establish how these features are mechanistically connected. The aim of the present study was therefore to probe the relationships between emerging behavioral dysfunctions with biological changes (molecular, cellular, regional atrophy) in male and female R6/2 mice.

## Materials and Methods

### Animals

All procedures were carried out according the Animals (Scientific Procedures) Act 1986 and were approved by King’s College London ethical review panel (Designation no PCD 70/2901). Hemizygous R6/2 mice were bred by backcrossing R6/2 males to (CBA×C57BL/6) F1 females (B6CBAF1/OlaHsd, Harlan Olac, Bicester, UK). Mice were genotyped and the CAG repeat size was measured as described previously [14]. The CAG repeat was 211.909±6.123 (SD) for males and 209.3±3.592 for females.

R6/2 and WT mice were housed under standard animal laboratory conditions. Temperature was automatically regulated at 21°C±1°C. Animals were kept on a 12 h light:dark cycle. Mice were group-housed dependent on gender, but genotypes were mixed within the cages. All mice had access to standard cage environmental enrichment (bedding and play tube). Mice were maintained on a standard chow diet with tap water available *ad libitum.* From 13 weeks of age, food was supplemented with a mash diet on the floor of the cages. To ease access to drinking water, elongated spouts were added to the water bottles. Body weight was monitored weekly from 6 weeks of age.

All procedures reported here (behavioral, MRI and histological) were conducted on the same cohort of animals. Littermates were divided into four groups: male wild type (WT, n = 9), male R6/2 (n = 11), female WT (n = 10) and female R6/2 (n = 10). An equal ratio between male:female was aimed for. For exact numbers of subjects included at each stage of analysis see Table S1.

### Behavioral Tests

#### Rotarod

The rotarod assesses motor ability and coordination. It is commonly used to chart the emergence and progression of motor deficits in animal models of HD. Mice were tested on a standard rotarod (Ugo Basile, Italy), with the modification of a smooth rubber coating over the rotating rod to minimize the mice from being able to cling to the beam. During each trial, mice were acclimatized to the apparatus by being placed on the rod rotating at the minimum speed of 4 rpm for 20 sec. Following acclimatization, the trial was initiated and the rod progressively accelerated from 4 rpm to 40 rpm over a period of 5 min. Latency (measured in sec) for mice to fall from the rod was recorded. For the first test session (acquired at 5 weeks of age), rotarod performance was tested 3 times per day for 4 consecutive days, but data for the first day was considered acclimatization to the test and thus not included in the final analysis. For the other two sessions (at 9 and 13 weeks of age), rotarod was performed over 3 consecutive days with 3 trials per day (data for the first acclimatization day was, again, not included in the analysis). The apparatus was thoroughly cleaned with 70% industrial methylated spirit (IMS) between testing each mouse.

#### Open field

Open field behaviors were tested at 5, 9 and 13 weeks of age. Mice were individually placed into a custom-built 100 cm diameter (35 cm deep) circular, white open field arena (Engineering & Design Plastics Ltd., Cambridge, UK) for 5 min to assess exploratory activity in a novel, unfamiliar environment. Therefore no habituation was given on this test. All behavior was recorded by a video camera positioned above the apparatus and analyzed later. The open field arena was thoroughly cleaned using 70% IMS between animals. Activity was assessed using EthoVision 7XT software (Noldus, Netherlands); distance moved throughout the trial was automatically traced and calculated. The open field arena was divided into two zones by a circle drawn 4 cm from the outer walls, thus creating an inner- and outer-zone. Thigmotaxis, the time spent in the peripheral, outer-zone of an open field is indicative of an anxiety-like behavior [27]. Thigmotaxis was measured by calculating a percentage of total time spent in the outer-zone over the entire trial. This measure was scored at least twice by two investigators who were blind to the treatment groups and until values were within ≥ 95% confidence.

#### Passive avoidance

It has been shown previously that R6/2 mice were deficient in memory performance at the passive avoidance task from 6 weeks of age [19]. To capture this potentially early behavior deficit, mice were assessed at 6 weeks of age, using an adapted protocol [28]. A step-through box was used with a light and dark compartment separated by an automated guillotine door (Gemini Avoidance System, SD Instruments, San Diego, USA). The paradigm was divided into two days: an initial “training” day followed 24 h later by the “testing” day. On the training day, mice were individually held in the light compartment with the connecting door in the open position. The time taken for each mouse to move into the dark compartment was recorded to determine any genotype-dependent differences in general motivation to explore this novel environment. Once the mouse entered the dark compartment, the guillotine door closed automatically and a single footshock (0.4 mA, 1 sec duration) was administered. Mice were then kept in the dark chamber for 10 sec before being removed and returned to the home cages. During the testing day (24 h following training), memory retention, expressed as latency to enter the dark chamber, was recorded. Mice were individually placed into the light compartment and latency to cross into the dark chamber was recorded out of a possible total of 5 min. If the mouse entered the dark chamber during the test day, the guillotine door closed and the trial was complete. If the mouse remained in the light compartment for the full duration of the test, it was removed after 5 min. No footshocks were administered during the testing day of the paradigm. On both the training and testing days, the passive avoidance equipment was thoroughly cleaned with 70% IMS between mice.

### Magnetic Resonance Imaging

Mice were anaesthetized using 5% isoflurane along with a combination of medical air (0.7 l/min) and oxygen (0.3 l/min). Once fully anaesthetized, mice were positioned and fixed into a plastic frame, where anesthetic was administered through a facemask. Mice were maintained under anesthesia, typically between 1–2% isoflurane for the duration of the scanning. Temperature was maintained through a homeostatic heating airflow system and breathing rate monitored through a respiration balloon positioned under the thorax (Small Animal Instruments, New York, USA). Post scanning, but prior to recovery, mice were administered 0.1 ml saline i.p. to abate dehydration.

Images were acquired on a 7T horizontal bore Magnetic Resonance Imaging (MRI) system (Varian, Paolo Alto, California, USA), with a 100 Gauss gradient set insert and a 39 mm-bore (transmission and receiver) radiofrequency coil (Rapid, Germany). The scanner was controlled through VnmrJ software (Varion, Paolo Alto, California, USA). Correct positioning of the mouse within the RF coil was confirmed through a series of scouting images. A Multi-Echo-Multi-Slice (MEMS) scan was then acquired (*TR* = 2500 msec, *TE* = 10 msec, echo train = 8, averages = 4, matrix = 128×128, FOV = 20×20 mm, 30 coronal slices at 0.5 mm thickness, 21 min acquisition time). Coronal slices were positioned based on a reproducible anatomical marker (the most visibly posterior part of the cerebellum).

Post-acquisition image processing was conducted using VnmrJ software prior to conversion to the ANALYZE 7.5 file format. All eight echoes were summed into a single structural image set. These images were used to manually delineate neuroanatomical structures (Fig. S1) in JIM Ver. 5.0 (Xinapse Systems, Alwincle, UK). Regions-of-interest (ROIs) consisted of: whole brain, cortex, striatum, hippocampus, and corpus callosum. ROIs were delineated by two investigators blinded to the experimental groupings with intra- and inter-rater reliability consistently ≥ 95% confidence level. All information outside of the ROIs was subsequently masked out, the ROIs were then individually saved in NIFTI format. Volumetric data were calculated and processed semi-automatically using Python Ver.2.6 (Python Software Foundation). To measure changes in T2 relaxivity (reflective of tissue composition), maps of T2 signal intensity were obtained through a mono-exponential fit of the eight echoes. The NIFTI files, created through delineating the ROIs onto the structural images, were superimposed onto the maps of T2 signal intensity allowing for the generation of mean T2 relaxation times within each ROI. A small circular ROI was taken for cheek muscle tissue T2 relaxivity in order to act as an internal control measure.

### Histology

Animals were sacrificed through terminal anesthesia with Euthatal (Marial, Harlow, UK) administered immediately following the final MR scan at 14 weeks of age. Brains were removed and fixed in 4% Parafix (Pioneer Research Chemical Ltd., Essex, UK) for 48 h. The brains were then rinsed in phosphate buffered saline (PBS) and stored in 30% sucrose, and 0.05% sodium azide, in PBS until sectioning. Coronal sections were taken serially at 50 µm thickness on a freezing microtome (HM430 Microm, Thermo Scientific), and stored at −20°C in tissue cryoprotective solution containing 0.05% sodium azide until staining.

#### Immunohistochemistry

Sections were washed in PBS prior to incubation for 30 min in 3% H_2_O_2_ in PBS to quench endogenous peroxidase activity. Non-specific binding was blocked with a 1 h incubation in 10% normal serum with 0.3% Triton X-100 in PBS. Sections were then incubated overnight at 4°C in primary antibodies against NeuN (1∶500, Millipore, Watford, UK) or S830 (1∶2000), raised against exon 1 HTT with 53 glutamines [29] prior to incubation in appropriate biotinylated secondary antibody (Vector, Peterborough, UK) for 2 h at RT and followed by 1 h incubation in an avidin-biotinylated-peroxide complex (1∶100, Vector, Northampton, UK). 3, 3′-diaminobenzidine (Sigma-Aldrich, Poole, UK) was used as the chromagen.

#### Cortical thickness

Assessment of regional cortical atrophy was determined by thickness measurements of primary motor cortex (M1) and primary sensory cortex (S1). In each region 10 vertical lines were drawn covering all layers from the most dorsal horn of the corpus callosum to the pial surface. The mean length taken was for 3 consecutive sections caudally from when the corpus callosum bridges across the two hemispheres (approximately Bregma 1.10 mm).

#### Stereology of NeuN-stained sections

Unbiased stereological estimates of volume and neuronal number were obtained using StereoInvestigator software (Microbrightfield, Willston, VT). All stereological measurements were performed with the observer being blind to the animals’ condition. The Cavalieri method was used to obtain unbiased estimates of striatal and M1 cortical reference volumes [30]. ROIs were defined at ×1.6 by reference to neuroanatomical landmarks. For both the striatum and M1 cortex, equally spaced sections (50 µm thickness each, 450 µm gap) were analyzed. As defined by Sadikot & Sasseville [31], sections contained within the striatum were sampled anteriorly from the first appearance of the genu of the corpus callosum (bregma = 1.1 mm) to posteriorly the first evidence of a hippocampal formation (bregma = −0.94 mm). The dorsal and lateral boundaries consisted of the corpus callusum with the medial boundary being the lateral ventricles/internal capsule. For sections rostral to where the dorsal 3^rd^ ventricle has joined the lateral ventricles, ventral boundaries become lateral ventricles/globus pallidus. The striatal volume was samples by 4–5 sections for both WT and R6/2. M1 cortex was measured anteriorly from 1.1 mm bregma to posteriorly −0.94 mm bregma from layers II to VI, as defined in a stereotaxic atlas [32]. The absence of cortical layer IV (indicative of the S1 cortex) defined the lateral boundaries of M1, whereas medial boundaries consisted of the most dorsal part of the corpus callosum. M1 was samples by 4–5 sections for both WT and R6/2.

To obtain unbiased estimates of neuronal numbers, the optical fractionator was employed as a stereological probe (coefficient of error <0.1). Section thickness and neuronal counts were performed under oil immersion with the x100 objective (Zeiss) with a numerical aperture of 1.4. A sampling grid was applied appropriate to the structure measured (cortex = 200 µm×200 µm, striatum = 400 µm×400 µm) with a counting frame of 65 µm×35 µm with a mean thickness of 18 µm. Guard zones of 0.5 µm were applied at the top and the bottom of each frame with a mean dissector height of 17 µm.

#### Quantitative analysis of S830

Evaluation of mHTT immunoreactivity in different brain regions was performed using an intensity-based measurement of S830 staining. Non-overlapping images (using fixed exposure and light intensities at ×40) were obtained from 3 consecutive sections expressing the striatum, cortex or hippocampus. In total, 30 striatal, 60 cortical and 36 hippocampal images were taken. All images were captured in RGB using a live video camera (JVC, 3CCD, KY-F55B), mounted onto a Zeiss Axioplan microscope.

Staining intensity was quantified using threshold-based analysis software (Image Pro Plus, Media Cybernetics, IL, USA) assessing optical density of the immunoreactive product. Threshold levels were chosen based on the minimum level of transmitted light needed to detect the immunoreactive product on a scale of 0 (100% transmitted light) and 255 (0% transmitted light) for each pixel. Two levels were taken to measure dense, nuclear mHTT inclusions (90), and total mHTT staining (nuclear and extra-nuclear, 130); mean percentage immunoreactivity area per field of view (FOV) was recorded.

### Statistical Analysis

All data were screened for statistical outliers using Grubb’s Test (GraphPad Software, California, USA). Due to animal loss, occasional missing data samples or statistical outliers, the number of animals varied for each test (Table S1). For data where repeated measures have been taken, such as the longitudinal behavioral and MRI data, these were analyzed using a two-way ANOVA with Time and Group as between-subject factors. For tests with a single time point of data acquired, either an unpaired *t*-test or two-way ANOVA was used where appropriate. Bonferroni’s post-hoc analysis was applied for multiple comparisons; main effects of statistical analyses are quoted in Table S2. The Pearson Correlation Coefficient was used for all correlative analyses. Due to the absence of a mHTT stain in WT, this marker was not included in the analysis. Statistical analyses were calculated using SPSS Statistics Ver.20 (IBM, Portsmouth, UK). Graphs were constructed using Prism Ver.5.0b (GraphPad Software, California, USA).

## Results

**Figure 1:**
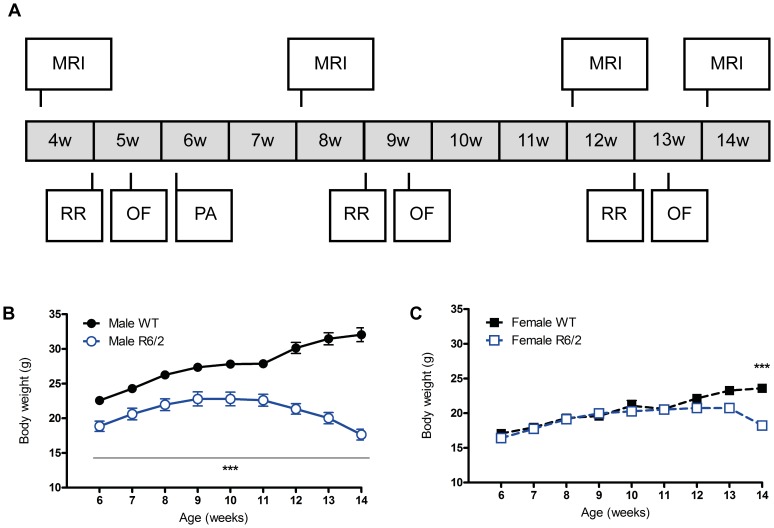
Experimental design and body weight of WT and R6/2 mice. (A) Experimental protocol. Mice were scanned for MRI measures at 4, 8, 12 and 14 weeks of age. The final MR scan was terminal, mice were culled and brains removed for histological analysis. Performance on a rotarod (RR) and behaviors expressed within an open field (OF) were assessed at 5, 9 and 13 weeks of age. Cognitive performance at the passive avoidance (PA) test was measured at 6 weeks of age only. (B & C) Assessment of body weight. Both male (B) and female (C) R6/2 mice exhibited progressive loss in body weight. This was highly significant from 6 weeks of age for males, but only reached statistical significance at 14 weeks of age for the females. Data presented as means ± SEM; ***p<.001.

The four groups of mice (male WT, male R6/2, female WT, female R6/2) underwent *in vivo* MRI scanning at 4, 8, 12 and 14 weeks of age (Fig. 1A). Rotarod performance and open field behaviors were assessed at 5, 9 and 13 weeks of age. Cognitive performance of mice in the passive avoidance test was assessed at 6 weeks of age only. After the final MRI session, animals were culled for post-mortem immunohistochemical analyses of neuropathological markers.

### Physiological Measures

A failure to gain weight and the development of muscle atrophy is a classic feature of HD [33]. An interaction of time and the four groups (F(Group×Time)_24,310_ = 7.175, p<.001) indicates that body weight gain of the four groups of mice was different over time. The weight of male R6/2 mice increased up to 9 weeks of age before gradually decreasing. Male R6/2 mice were consistently lighter compared to their WT littermates even at 6 weeks of age (p<.001, Fig. 1B). In contrast, there was no significant difference in body weight between WT and R6/2 female mice until 14 weeks of age (p<.001, Fig. 1C). Importantly, both male and female R6/2 did not differ in the number of CAG repeats. These results indicate that there are gender-dependent, as well as genotype-specific effects that are reflected in the phenotype of these animals. These effects were also evident in the development and the progression of the performance of R6/2 mice on behavioral measures compared to WT.

### Onset of Behavioral Deficits in R6/2 Mice is Gender-dependent

**Figure 2:**
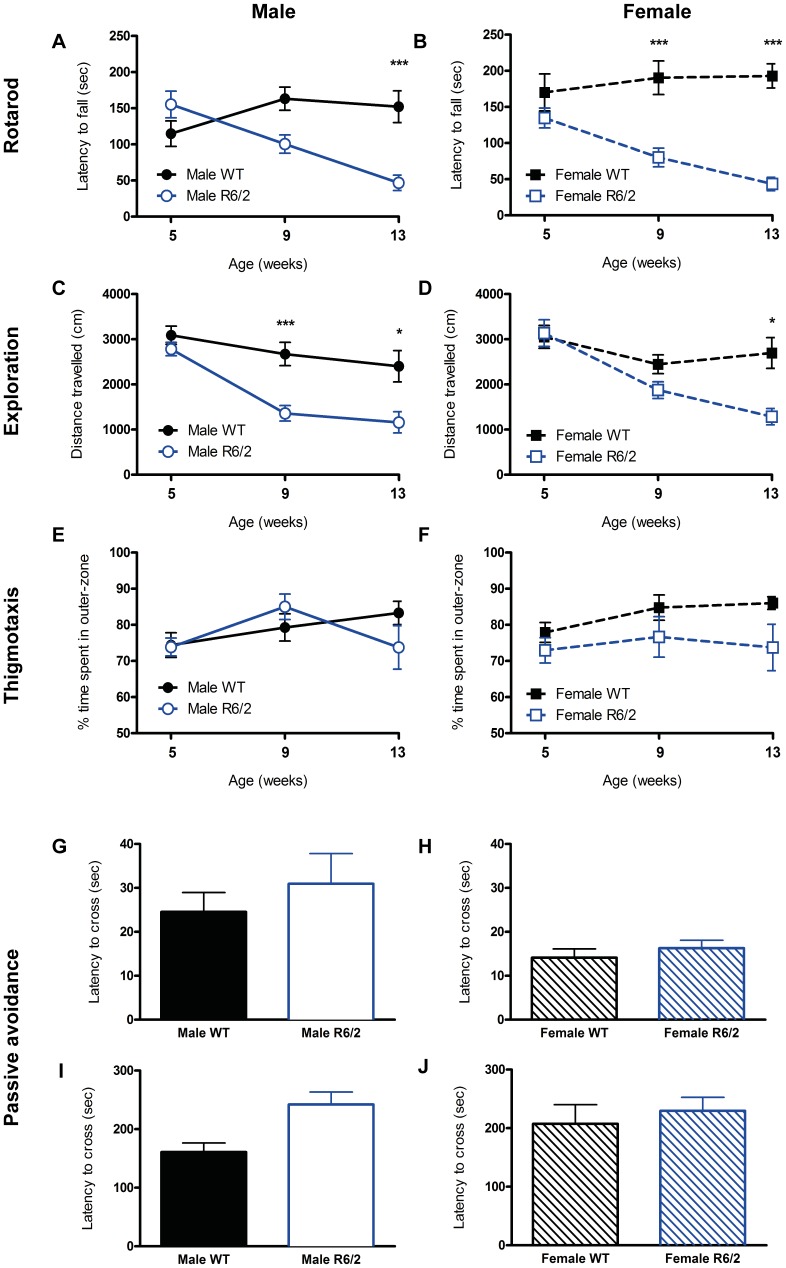
Behavioral characterization of WT and R6/2 mice. (A & B) R6/2s exhibited a decline in latency to fall from the rotarod beam with age, reaching statistical significance from 9 weeks of age for the female mice, but only at 13 weeks of age for the males. (C – F) R6/2 also developed age-related diminished exploration in an open field, detectable from 9 weeks for the males, but at 13 weeks only for the females, however, there were no changes in time spent in the periphery of the arena (thigmotaxis). (G – J) There was no difference in passive avoidance training or memory in 6 week old R6/2 mice. Data presented as means ± SEM; *p<.05, ***p<.001.

On the rotarod, male (Fig. 2A) and female R6/2 (Fig. 2B) exhibited a significant linear decline in performance from 5 to 13 weeks of age (F(Group)_3,102_ = 18.794, p<.001). However, at 5 weeks of age, male R6/2′s latency to fall was 26.01% longer than that of the WT, indicating a better, albeit non-significant, performance on this task at an early age. In comparison, female R6/2 at 5 weeks of age already exhibited an emerging deficit. Significant deficits were apparent from 9 weeks of age for female R6/2 (p<.001), whereas male R6/2 were only significantly impaired at 13 weeks of age (p<.001). Female R6/2 therefore exhibited earlier evidence of impairments in sensorimotor coordination compared to male R6/2. For both genders, performance decreased by 73.72% compared to WT by 13 weeks of age. However, this deficit cannot be explained by a change in overall activity, as general exploration (in an open field, F(Group×Time)_6,103_ = 2.439, p = .03) was reduced earlier in male R6/2 at 9 weeks of age (Fig. 2C, p<.001) compared to 13 weeks of age for female R6/2 mice (Fig. 2D, p = .018). This emergence of diminished exploratory behavior was not accompanied by anxiety in these animals, as neither male, nor female, R6/2 exhibited changes in thigmotaxis compared to WT (F(Group)_3,101_ = 2.274. p = .085; Fig. 2E&F). R6/2 also did not enter the dark chamber on passive avoidance training faster than controls at 6 weeks of age (F(Genotype)_1,34_ = .818, p = .372; Fig. 2 G&H), further indicating that there was no evidence of altered anxiety expressed at this age that could account for the development of decreased exploratory locomotor behavior. Despite an overall difference between genotypes (F(Genotype)_1,36_ = 4.527, p = .04), memory performance on the testing of passive avoidance was also comparable to controls when corrected for multiple comparisons, hence implying that these cognitive functions were not affected at 6 weeks of age (Fig. 2 I&J).

**Figure 3:**
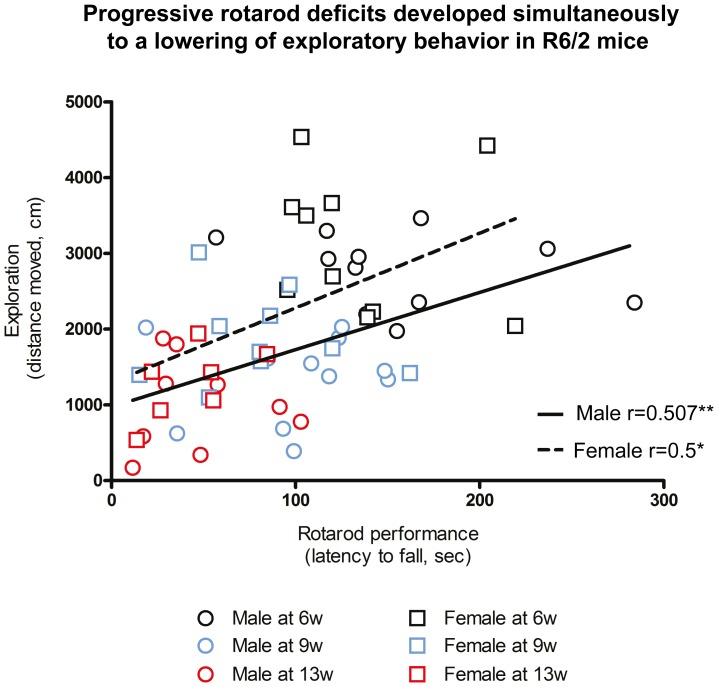
Progressive rotarod deficits developed simultaneously to a lowering of exploratory activity in R6/2 mice. Latency to fall from an accelerating rotarod against exploratory activity measured in an open field for all R6/2 mice; data points were separated into the three ages investigated, at 5, 9 and 13 weeks of age. Positive correlations were detected between these two tasks over time due to the simultaneous development of deficits in both male (r = 0.507, **p<.01) and female (r = 0.5, **p<.01) R6/2.

Although there was a gender-specific onset of deficits on the rotarod and lower exploration in the open field for the R6/2 mice, both measures were correlated (Table S3), potentially indicating that performance on both tasks decreased following a similar pattern (Fig. 3). Importantly though, as performance progressively decreased on different tasks, a funnel effect occurs that gradually shifts toward their origin (i.e. 0). This reduces variability in affected tasks and is more likely to reflect a common cause than both tasks influencing each other directly. Indeed, rotarod and exploration were not correlated at 6 weeks of age (Table S4), but as both deficits worsened, the relationship between both tasks increased (Fig. S2). Rotarod and exploratory deficits therefore developed in a genotype- and gender-specific pattern, but no significant deficits in anxiety or memory were evident. Although there is an emerging connection between rotarod and exploration, this link is likely due to a common underlying cause, such as brain atrophy.

### Atrophy and Changes in Tissue Characteristics in R6/2 Mouse Brain are Genotype- and Gender-dependent

**Figure 4:**
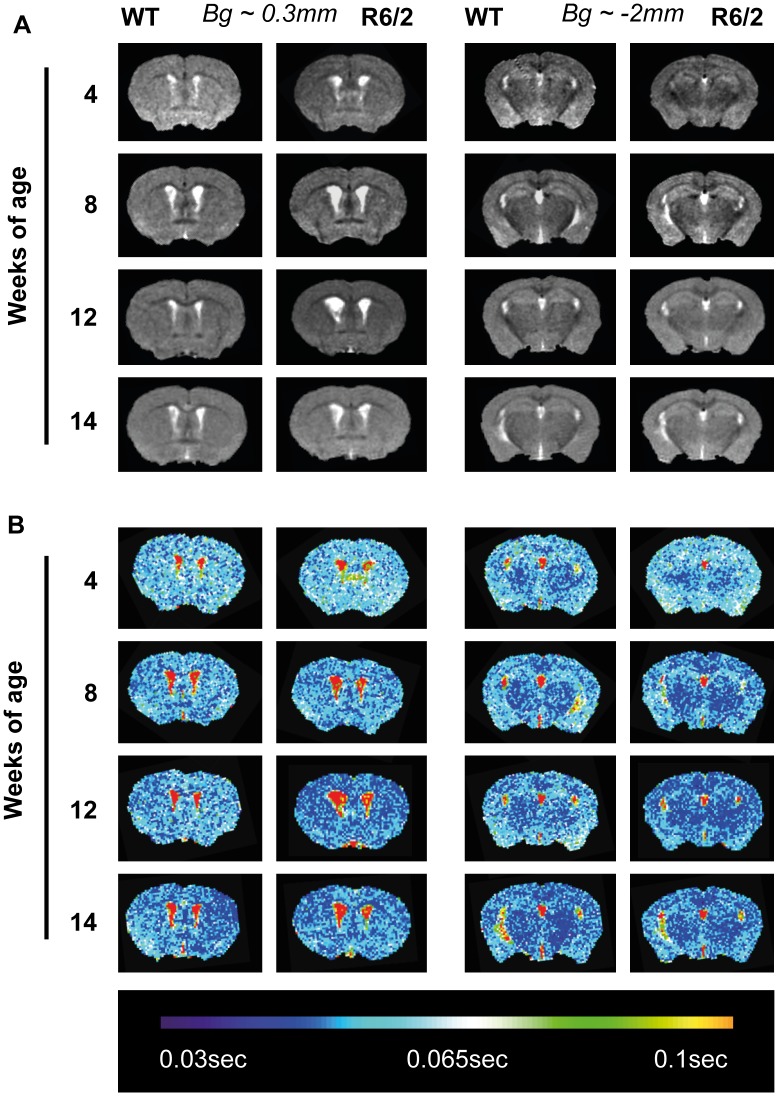
*In vivo* MRI sample images. (A) Sample T2-weighted images for structural volumetry assessments. Images are from scans taken at 4, 8, 12 and 14 weeks of age at anatomically matching slices. (B) Hot/cold scaled maps of T2 relaxation times for the study of T2 relaxivity, these are represented on anatomically identical images to those in (A). Bg = bregma.

**Figure 5:**
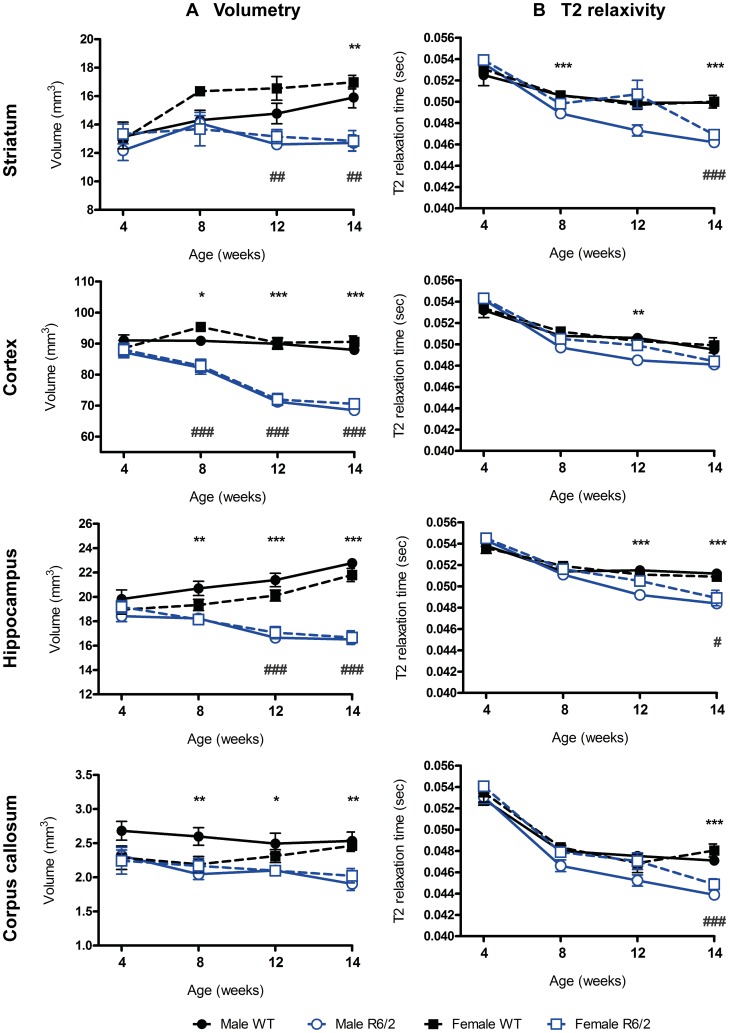
MRI assessment of brain volumetry and T2 relaxivity in WT and R6/2. (A) Regional brain volumes assessed through MRI (volumetry) became progressively smaller in both male and female R6/2 mice versus WT, with the exception of the female R6/2 corpus callosum, which did not differ as compared to WT at any age. (B) T2 relaxation times (T2 relaxivity) progressively shortened for both WT and R6/2 with age, but this was significantly exacerbated in the R6/2 in all regions of interest investigated. Data presented as means ± SEM; males: *p<.05, **p<.01, ***p<.001, females: ^#^p<.05, ^##^p<.01,^ ###^p<.001.

Concomitant to behavioral assessments, longitudinal MRI was used to non-invasively interrogate neuroanatomy (Fig. 4A). A manual delineation of neuroanatomical structures (Fig. S1) indicated that immediately post-weaning (4 weeks of age), there were no genotype or gender differences in brain volumetry (Fig. 5A). Nevertheless, by 8 weeks of age cortex (F(Group×Time)_9,137_ = 7.345, p<.001) and hippocampus (F(Group×Time)_9,137_ = 7.727, p<.001) were on average 10.22% smaller in R6/2 than WT. Interestingly, only male R6/2 mice exhibited smaller (p<.05) corpus callosum volumes (F(Group×Time)_9,134_ = .896, p = .531). A decreased whole brain volume (F(Group×Time)_9,137_ = 6.384, p<.001) also reflected these widespread changes (data not shown). This trend continued with major atrophy evident throughout the brain at 14 weeks of age. A comparatively (20.47%) smaller volume of the female R6/2 striatum (F(Group)_3,137_ = 13.77, p<.001) was already apparent at 12 weeks of age (p = .003). Importantly though, neither striatal nor hippocampal volume, in the R6/2 shrunk, but instead they remained stable in size compared to an increase in volume in WT. Interestingly, for male R6/2, cortical volume changes were significantly correlated with changes in other brain regions, whereas the striatum was not; a similar, but less robust effect, was also observed in females (Table S5).

These structural effects were accompanied by changes in T2 relaxivity (Fig. 4B), which reflect alterations in tissue characteristics (Fig. 5B). T2 relaxivity dropped by almost 7% on average between 4 and 8 weeks of age in all animals, indicating a general maturation effect, all brain ROIs investigated exhibited a significant (p<.05) Group×Time interaction (Fig. S2). At 12 weeks of age, R6/2 males exhibited a more significant shortening of T2 in the cortex (p = .001) and hippocampus (p<.001), suggesting an interaction between genotype and gender. By 14 weeks, this T2 shortening was also apparent in female R6/2. All brain T2 relaxivity measures were correlated with each other and even cheek-muscle, albeit to a lesser extent (Table S5). Unlike brain region volumes, tissue T2 values were generally highly correlated with each other from as early as 4 weeks of age (Table S6). Relaxivity changes in R6/2 mice therefore might indicate a more fundamental change in tissue characteristics. However, R6/2 cheek-muscle T2 values did not significantly differ between the four groups over time (F(Group×Time)_9,136_ = .648, p = .754), or when compared to controls at any time point studied. The largest difference was observed at 14 weeks (WT = 0.031±0.002 msec versus R6/2 = 0.029±0.002). Interestingly, cortical volume was associated with T2 changes in all brain regions, potentially suggesting that cortical atrophy is linked to global changes in tissue characteristics. Overall, these results demonstrate that, similar to the behavioral phenotype, changes in brain atrophy and tissue characteristics in R6/2 are generally progressive with subtle gender differences.

### Cortical Abnormalities are Associated with Motor Deficits

The neuroanatomical basis of behavioral impairments is reflected in associations between these two outcome measures. Both rotarod performance and exploratory activity were generally more associated with T2 relaxivity than volumetry (Table S7). Interestingly, striatal volume was not associated with behavioral measures, whereas cortical atrophy and T2 relaxivity were correlated with decreased performance in both rotarod and lower levels of exploration in the open field. Neuroanatomical changes were generally not associated with cognitive measures, such as thigmotaxis. Indeed, only hippocampal volume (and whole brain volume) in male R6/2 was related to thigmotaxis. Overall, the stronger correlations between behavior and T2 relaxivity potentially reflect the importance of tissue characteristics rather than mere volume assessment. Importantly, behavioral deficits and neuroanatomical changes started to emerge between 5 and 8 weeks of age and progressively declined thereafter. As neuroanatomical structures changed and T2 relaxivity decreased with time, the associations with motor performance across both WT and R6/2 mice generally increased (Table S8). This intensification of associations may reflect the increasing downstream influence the molecular pathology exerts on brain structure, as well as on behavioral outcome measures.

### Molecular Pathology in the Absence of Neuronal Loss

**Figure 6:**
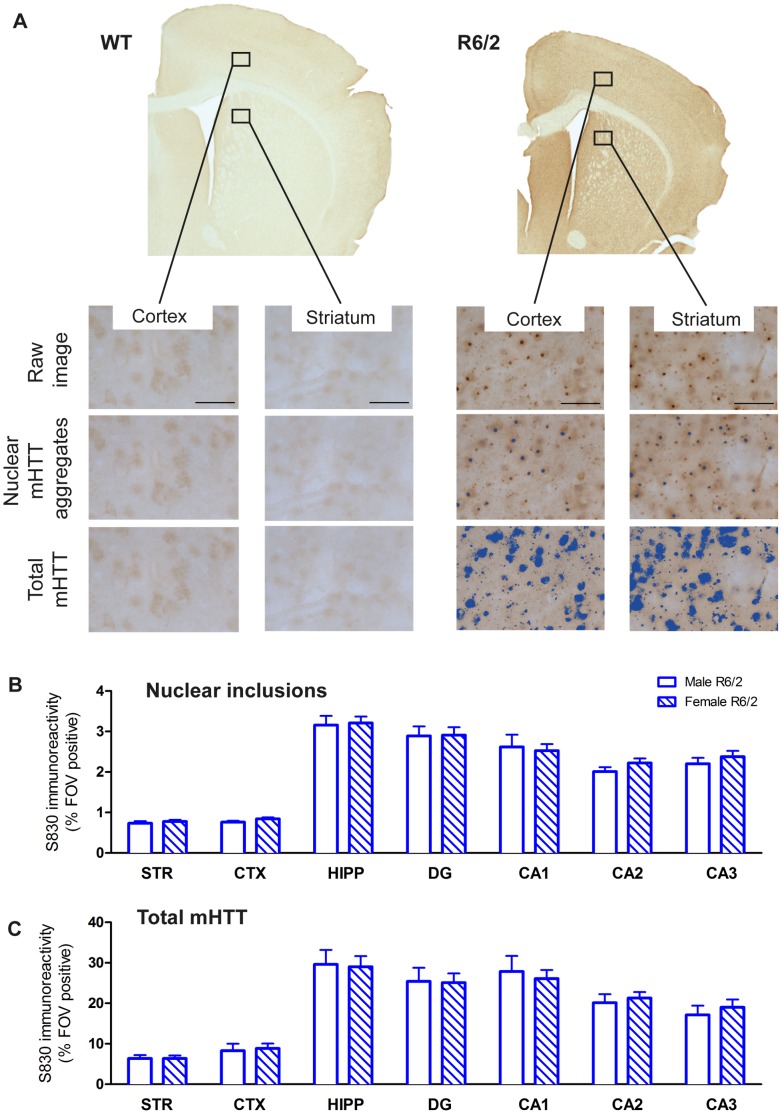
Quantification of mHTT accumulation. (A) Representative coronal sections from 14 weeks old WT and R6/2 mouse brains stained with the S830 antibody for the detection of mHTT. Levels were quantified within seven brain regions using an intensity threshold-based image analysis tool optimized for the detection of nuclear inclusions, or total levels of mHTT (neuropil aggregates and diffuse nuclear accumulation, highlighted in blue); scale bar 50 µm. Percentage of sampled field of views (FOVs) positive for S830 stain of nuclear inclusions (B), and percentage of FOV positive for total mHTT (C); there were no significant differences between male and female R6/2 for either assessments. Regional differences in mHTT reflect cellular density. STR = striatum, CTX = cortex, HIPP = hippocampus, DG = dentate gyrus, CA1 = hippocampal CA1 subfield, CA2 = hippocampal CA2 subfield, CA3 = hippocampal CA3 subfield. Data presented as means ± SEM.

The deposition of mHTT is a neuropathological hallmark of HD [34], [35]. We used the S830 antibody to independently visualize nuclear inclusions and other forms of aggregated mHTT in both the nucleus and neuropil (referred to as total mHTT) in the R6/2 mouse brain (Fig. 6A). Due to the limited variation in CAG repeats, there was no correlation between CAG repeat size and nuclear inclusions or total mHTT levels. As these mHTT deposits were not observed in WT animals (WT cortex = 0.347±0.2% FOV immunoreactive; striatum = 0.095±0.0382%, hippocampus = 0.551±0.44%), this pathology is specific to the R6/2 genotype. There was no gender-dependent difference in either the nuclear inclusion or total mHTT load (Fig. 6B&C). Levels of total mHTT were highly correlated across brain regions (Table S9), with the exception of total mHTT in the striatum of female R6/2 mice which did not correlate with this measure in any other region. Nuclear inclusion levels in R6/2 males, but not in females, were also widely correlated with total mHTT across brain regions. These findings indicate a ubiquitous abundance of mHTT across various brain regions. Importantly though, associations in female R6/2 indicated a general lack of associations for nuclear inclusions between different brain regions, as well as a specific lack of correlations between total mHTT levels in the striatum with all other regions.

**Figure 7:**
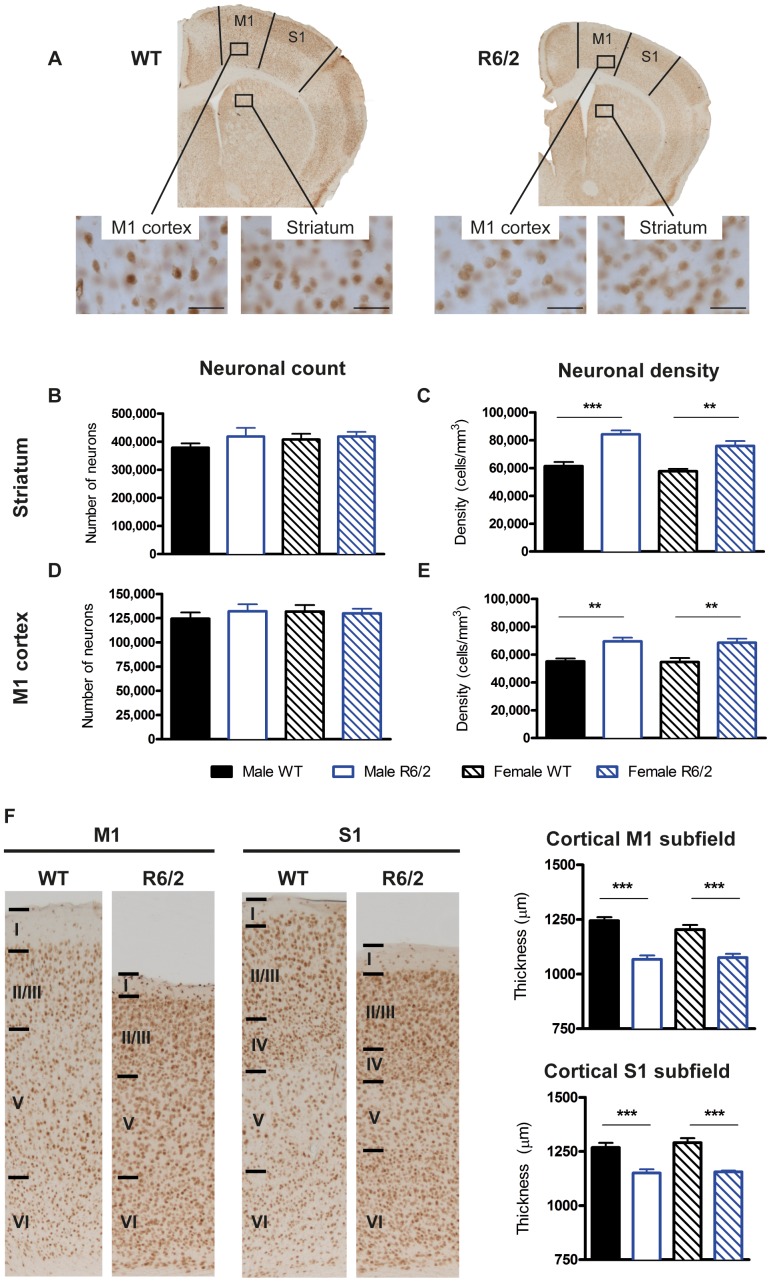
Histological characterization of R6/2 neuropathology on NeuN stained sections. (A) Representative coronal sections taken from 14 weeks old WT and R6/2 brains stained for NeuN; scale bar 50 µm. A stereological analysis revealed there were no differences in neuronal number in either the striatum or primary motor (M1) cortex (B & D), but there were increases in neuronal density in both these areas (C & E). There was a dramatic reduction in both the M1 and somatosensory (S1) cortical thickness in the R6/2 s (F). Data presented as means ± SEM; **p<.01, ***p<.001.

A stereological analysis of sections, stained for the neuronal marker NeuN (Fig. 7A), nevertheless, found that there was no decrease in the number of neurons in the striatum (F(Genotype)_1,30_ = 1.119, p = .299; Fig. 7B) or motor (M1) cortex (F(Genotype)_1,27_ = .204, p = .655; Fig. 7D). In the absence of neuronal loss, tissue shrinkage (on average 18.12%) resulted in a significant (p<.01) increase in neuronal density in both striatum (F(Genotype)_1,29_ = 53.341, p<.001; Fig. 7C) and M1 cortex (F(Genotype)_1,27_ = 27.784, p<.001; Fig. 7E). In the M1 cortex, this increase in neuronal density was accompanied by a highly significant thinning of both the M1 (F(Genotype)_1,31_ = 67.164, p<.001; Fig. 7F) and somatosensory (S1) cortex (F(Genotype)_1,30_ = 41.368, p<.001). As expected, neuronal number and density was highly correlated in both WT and R6/2 mice, but this effect was greater in the striatum as compared to the M1 cortex (Table S10). Interestingly, the number of neurons in the striatum and cortex were linked to their respective volumes in male but not female R6/2 mice. Histologically, there were very few associations between neuronal number or density with mHTT levels within any given brain region (correlations not shown). A higher level of cortical nuclear inclusions related to higher neuronal density in the M1 cortical subfield of female R6/2 (r = 0.94, p<.01) and to higher M1 cortical neuronal number for both male and female R6/2 s (r = 0.533, p<.05). Thus, R6/2 mice did not exhibit neuronal loss and levels of mHTT in the R6/2 brain did not appear to affect neuronal cell counts.

### Molecular and Cellular Pathology Correlate with Changes in Brain Structures

MRI detected substantial brain abnormalities in R6/2 mice. Correlating these results with neuropathological markers may reveal how system changes in brain structures provide a connection between molecular and cellular changes with the emergence of behavioral abnormalities. In R6/2 females, there appeared to be non-significant correlations between mHTT levels in the striatum and age-matched T2 relaxivity measures in the different brain regions (Table S11). In contrast, in male R6/2 only, a smaller cortical volume was associated with higher levels of nuclear inclusions and to a lesser degree with total mHTT (non-significant). A distinction between nuclear inclusions and total mHTT therefore provided a gender-specific association of molecular pathology with changes in brain structures. Stereological measurements of cellular and volumetry changes indicated a connection between brain-specific T2 relaxation values in female R6/2 with M1 cortical volume (Table S12). Across WT and R6/2 mice, MRI brain volumes were associated with neuronal density; neuronal density is therefore associated with changes in regional brain volumes assessed by MRI, but there is no correlation with total number of neurons within these regions.

### Associations of Molecular and Cellular Pathology with Behavioral Deficits

Combining assessments of histopathological burden and end-stage behavioral performance will provide information as to the association of the molecular and cellular pathology with behavioral abnormalities. However, there were few associations between the presence of mHTT and the extent of end-stage behavioral abnormalities exhibited by the R6/2 (correlations not shown); indeed, the only significant correlations were detected for female R6/2, where a poorer rotarod performance was associated with lower total mHTT levels in the hippocampal CA3 (r = 0.845, p<.05), and higher levels of nuclear inclusions were related to more exploratory behavior in the open field (r = -0.891, p<.05). There were, however, comparably more associations between stereological measures and behavioral performance (Table S13). Unexpectedly, for male R6/2, a worse performance on the rotarod was associated with higher neuronal number, density and volume of the striatum. No such effects were detected in the M1 cortex. These results imply a complicated relationship between molecular and cellular pathology with behavioral changes.

## Discussion

Establishing how molecular pathology in Huntington’s disease (HD) leads to structural brain changes and the emergence of behavioral abnormalities remains a major challenge. Here, we describe how a longitudinal assessment of behavioral performance and regional brain changes, as well as molecular and cellular pathological markers can inform on how these distinct measures are associated with each other. The principal findings were that (1) R6/2 developed progressive behavioral impairments that were influenced by gender and associated with changes in brain structures as measured by longitudinal MRI; (2) despite dramatic brain shrinkage and widespread deposition of mHTT, there was no neuronal loss in the regions investigated; (3) MRI detected regional brain abnormalities were associated with molecular and cellular pathology, as well as behavioral deficits. Regional brain changes hence provide an intermediary non-invasive biological measure that putatively can be used to predict changes in molecular/cellular pathology and its potential impact on impairments.

### Gender-dependent Differences in R6/2 Phenotype

We have detected a gender-dependent temporal presentation of various pathological phenotypes that could not be ascribed to differences in CAG repeat length, which was well-matched between R6/2 males and females. Gender effects in unconditioned behaviors (e.g. climbing, rearing, walking) in R6/2 and the *Hdh*Q140 knock-in mice have been previously noted, but these phenomena did not extend to rotarod performance [36], [37], [38]. Although we identified a gender-effect on rotarod performance in this study, this is not consistent with our previous observations and may be a reflection of a generally lower male WT performance. However, exploratory activity did appear to decline earlier in male R6/2, which was a reflection of age of onset and progression, as ultimately all animals exhibit a similarly stable, substantial deficit at 13 weeks of age. Behavioral analysis of a rat transgenic model of HD found that although both sexes expressed certain aspects of disease-like phenotypes, only males developed a robust motor coordination deficit [39]. The motor deficits exhibited by male HD rats were associated with a loss and atrophy of striatal medium spiny neurons (MSNs) and with lower 17beta-estradiol plasma levels. Given that MSNs expressed both alpha- and beta-estrogen receptors, this could account for the gender differences that were noted. However, overall, evidence for gender effects in HD is sparse both in preclinical models and in the presentation of HD in the clinic.

### R6/2 Exhibit Molecular Pathology without Neuronal Loss

As expected, the deposition of mHTT was ubiquitous throughout the R6/2 brain and was not influenced by gender suggesting that any potential gender-effects on phenotype onset and progression are likely to occur downstream of mHTT pathology. Given that the CAG repeat size was highly comparable between the R6/2 mice used in this study, it was not possible to resolve any potential correlation between CAG repeat size and mHTT accumulation in the brain regions studied.

Despite the widespread distribution of mHTT in the brain, we found no evidence of neuronal loss in either the striatum or M1 cortex. This is in contrast to a previous report of a reduction of 25% of striatal neurons at 13 weeks of age [40], [41], possibly a reflection of the difference in CAG repeat size between the two R6/2 colonies. However, this could also be a reflection of differences in strain background, housing conditions and the protocols used for data collection. The extent of neuronal cell death in the striatum, when detectable, is still considerably lower than that which occurs in the human disease, which may be attributable to the comparably short life span of these mouse models. However, there was a clear increase in neuronal density accompanied by T2 shortening, indicating that alterations in tissue characteristics occur potentially prior to any neuronal loss. Changes in neuronal morphology [42], as well as non-neuronal cellular changes in the R6/2, such as an age-related decrease in microglia [43] and extracellular matrix molecules [44] could account for tissue atrophy prior to neuronal loss. The development of motor deficits in R6/2 mice reported here is therefore not a consequence of neuronal loss in the striatum or M1 cortex. Indeed, it is likely that such behavioral dysfunction is a consequence of abnormal activity observed in the neuronal circuitry of R6/2 mice [34], [41].

### Establishing an Association between Molecular Pathology and the Emergence of Behavioral Deficits

A correlation analysis between behavioral and biological measures can provide insights as the extent to which certain markers, and their development, are associated. For instance, motor onset in HD can be predicted by striatal volume [45] indicating that changes in striatal atrophy are related to motor symptoms. Despite a small striatum in R6/2 animals, there was no progressive atrophy that correlated with the onset or progression of motor deficits. In contrast, as previously reported [26], cortical and whole brain volume changes correlated with changes in motor performance. Longitudinal changes in cortical volume precede structural differences in striatum in R6/2, consistent with previous reports [25], [26]. These early cortical changes can potentially be explained by neuronal aggregates that appear first and accumulate faster in cortical regions as compared to the striatum of R6/2 mice [32], [46]. This deposition of mHTT has occurred prior to 4 weeks, an age at which we were unable to detect any structural abnormalities, indicating a delay in the appearance of volumetric changes.

Changes in tissue characteristics, as measured by MR relaxivity, indicated a shortening of brain T2 values that was evident by 8 weeks of age for both WT and R6/2 mice. This is may reflect ongoing tissue maturation, such as myelination [47], although a similar pattern of change was detected in muscle. Interestingly, in comparison to WT, T2 values for male R6/2 in the striatum exhibited the earliest changes (8 weeks) in the absence of a significant difference in volume. In contrast, atrophy in the cortex preceded T2 changes. Shortening of grey matter T2 values has also been described in HD patients [48] and correlated with symptom intensity [49]. These T2 relaxation changes may be attributable to the known increase in brain iron levels in HD patients [50], [51], [52], [53], [54] and R6/2 mice [55]. As suggested by the temporal dynamics, T2 changes were not directly correlated with neuronal density or mHTT aggregation. Indeed, it is likely that mHTT accumulation precedes increases in transition metal deposits [54]. However, it remains unclear how T2 values relate to these molecular and cellular abnormalities.

Previous evidence of a direct connection between mHTT deposition and the extent of disease phenotype in R6/2 mice is based on temporally matched abnormalities in neuronal/synaptic function [32], [56] and the development of behavioral defects [46]. However, a direct correlation or causal association between biological markers and behavioral phenotypes remain poorly documented. We have here demonstrated that longitudinal MRI measurements together with behavioral assessments have the potential to provide novel insights as to how these relate to each other in animal models of HD. Applying this methodology to the R6/2 mouse is challenging, given the very rapidly developing and widespread pathology throughout the brain. We expect that it will be more informative when applied to HD mouse models in which the disease phenotypes progress more slowly. Analysis of the pre-manifest and early stages of the disease will be key to establish which changes are causally related, as during the final stage of the disease all measures are intertwined. Still, more extensive longitudinal, as well as cross-sectional, studies would be required to provide a thorough understanding of how mHTT leads ultimately to behavioral dysfunction.

## Supporting Information

Figure S1 **Region of interest delineation criteria used for MRI analysis.** (A) Definition of criteria by which regions of interest were delineated onto structural MR images; quoted Bregma reference marks are typically ±0.25 mm as a result of the inconsistency of slice positional matching upon image acquisition. (B) Sample regions of interest delineated onto a 14 week WT mouse brain.
(TIF)

Figure S2 **Age related separation of rotarod performance and exploratory activity for WT and R6/2 mice.** Latency to fall from a rotarod versus exploration in an open field for both WT and R6/2 at the three behavioral time points, 5, 9 and 13 weeks of age. As the R6/2 s developed age-related deficits at both tasks, the WT and R6/2 data points separated creating significant, positive correlations for both genders when considering all animals together. *p<.05.
(TIF)

Table S1 Number of animals used. All tests were conducted on the same cohort of animals. However, there was a variable number of animals for the analysis of each test due to either death during the study, to the occasional missing data sample or to the exclusion of statistical outliers. RR = rotarod, EXP = exploratory activity, THG = thigmotaxis, PA (d1,d2) = passive avoidance (day 1, day 2), STR = striatum, CTX = cortex, HIPP = hippocampus, CC = corpus callosum, WB = whole brain, MUSC = muscle, DG = dentate gyrus, CA1 = hippocampal CA1 subfield, CA2 = hippocampal CA2 subfield, CA3 = hippocampal CA3 subfield, No. = neuronal number, Dens. = neuronal density, Vol. = volume assessed through stereology, M1 CTX/M1 = M1 cortex, S1 = S1 cortex.
(XLSX)

Table S2 Main effects derived from statistical analyses. Main effects derived from two-way ANOVAs. Time = repeated time of testing, Group = different experimental groups, G’type = genotype, Sex = gender. RR = rotarod, EXP = exploratory activity, THG = thigmotaxis, PA (d1,d2) = passive avoidance (day 1, day 2), STR = striatum, CTX = cortex, HIPP = hippocampus, CC = corpus callosum, WB = whole brain, MUSC = muscle, M1 CTX/M1 = M1 cortex, S1 = S1 cortex.
(XLSX)

Table S3 Correlation of behavioral measures over time. Correlations of performance at behavioral tasks, presented as Pearson r values. RR = rotarod, EXP = exploratory activity in an open field, THG = thigmotaxis in an open field. *p<.05, **p<.01.
(XLSX)

Table S4 Correlation of behavioral measures across the three time points investigated. Performance at longitudinal behavioral tasks correlated across the three ages investigated, 5, 9 and 13 weeks, presented as Pearson r values. RR = rotarod, EXP = exploratory activity in an open field, THIG = thigmotaxis. *p<.05.
(XLSX)

Table S5 Correlations of MRI measures of brain abnormalities over time. Correlation of all volumetry and T2 relaxivity measures across the regions of interest studied, presented as Pearson r values. STR = striatum, CTX = cortex, HIPP = hippocampus, CC = corpus callosum, WB = whole brain, MUSC = muscle. *p<.05, **p<.01.
(XLSX)

Table S6 Correlation of MR measures of brain abnormalities across the four time points investigated. Correlation of assessments of volumetry and T2 relaxivity in the regions of interest across the four ages investigated, 4, 8, 12 and 14 weeks, presented as Pearson r values. STR = striatum, CTX = cortex, HIPP = hippocampus, CC = corpus callosum, WB = whole brain, MUSC = muscle. *p<.05, **p<.01.
(XLSX)

Table S7 Correlation of behavioral decline versus MRI measures of brain abnormalities over time. Correlation of behavioral abnormalities and regional brain abnormalities determined through MRI, presented as Pearson r values. RR = rotarod, EXP = exploratory activity in an open field, THG = thigmotaxis in an open field, STR = striatum, CTX = cortex, HIPP = hippocampus, CC = corpus callosum, WB = whole brain, MUSC = muscle. *p<.05, **p<.01.
(XLSX)

Table S8 Correlation of behavioral performance against MR measures of pathology at three time points. Correlation of behavioral performance with age-matched assessment of brain abnormalities through MRI; 4 week MRI versus 5 week behavior, 8 week MRI versus 9 week behavior, 12 and 14 week MRI versus 13 week behavior. Presented as Pearson r values. RR = rotarod, EXP = exploratory activity in an open field, THIG = thigmotaxis, STR = striatum, CTX = cortex, HIPP = hippocampus, CC = corpus callosum, WB = whole brain, MUSC = muscle. *p<.05, **p<.01.
(XLSX)

Table S9 Correlation of regional levels of R6/2 brain mHTT. Correlation of regional levels of both total mHTT and nuclear inclusions across different brain regions in the R6/2, presented as Pearson r values. Tot mHTT = total mHTT, Nuc mHTT = nuclear mHTT inclusions, STR = striatum, CTX = cortex, HIPP = hippocampus, DG = dentate gyrus, CA1 = hippocampal CA1 subfield, CA2 = hippocampal CA2 subfield, CA3 = hippocampal CA3 subfield. *p<.05, **p<.01.
(XLSX)

Table S10 Correlation of measures of neuronal characteristics assessed through stereology. Correlation of stereological measures taken from NeuN-stained sections, presented as Pearson r values. STR = striatum, M1 CTX = M1 cortex, Neur no. = neuronal number, Neur dens. = neuronal density. *p<.05, **p<.01.
(XLSX)

Table S11 Correlation of mHTT levels against MRI measures of R6/2 brain pathology. Correlation of MRI measures of brain abnormalities taken at 14 weeks against mHTT abundance in R6/2 only, presented as Pearson r values. Tot mHTT = total mHTT, Nuc mHTT = nuclear mHTT inclusions, STR = striatum, CTX = cortex, HIPP = hippocampus, CC = corpus callosum, WB = whole brain, MUSC = muscle. *p<.05.
(XLSX)

Table S12 Correlation of neuronal characteristics versus MRI measures of brain abnormalities. Correlation of brain abnormalities at 14 weeks assessed through MRI against stereological analyses of NeuN-stained sections, presented as Pearson r values. STR = striatum, M1 CTX = M1 cortex, Neur no. = neuronal number, Neur dens. = neuronal density, CTX = cortex, HIPP = hippocampus, CC = corpus callosum, WB = whole brain, MUSC = muscle. *p<.05, **p<.01.
(XLSX)

Table S13 Correlation of behavioral measures against stereological assessments of neuronal characteristics. Correlation of behavioral performance at 13 weeks of age against stereological measures of neuronal characteristics on NeuN-stained sections, presented as Pearson r values. RR = rotarod, EXP = exploratory activity in an open field, THG = thigmotaxis in an open field, Neur no. = neuronal number, Neur dens. = neuronal density. *p<.05, **p<.01.
(XLSX)
