# Supplementary figures and images for: Correlations of Behavioral Deficits with Brain Pathology Assessed through Longitudinal MRI and Histopathology in the R6/2 Mouse Model of HD
Source: PLoS One. 2013 Apr 4;8(4):e60012. doi: 10.1371/journal.pone.0060012 (PMC3617160; doi:10.1371/journal.pone.0060012)

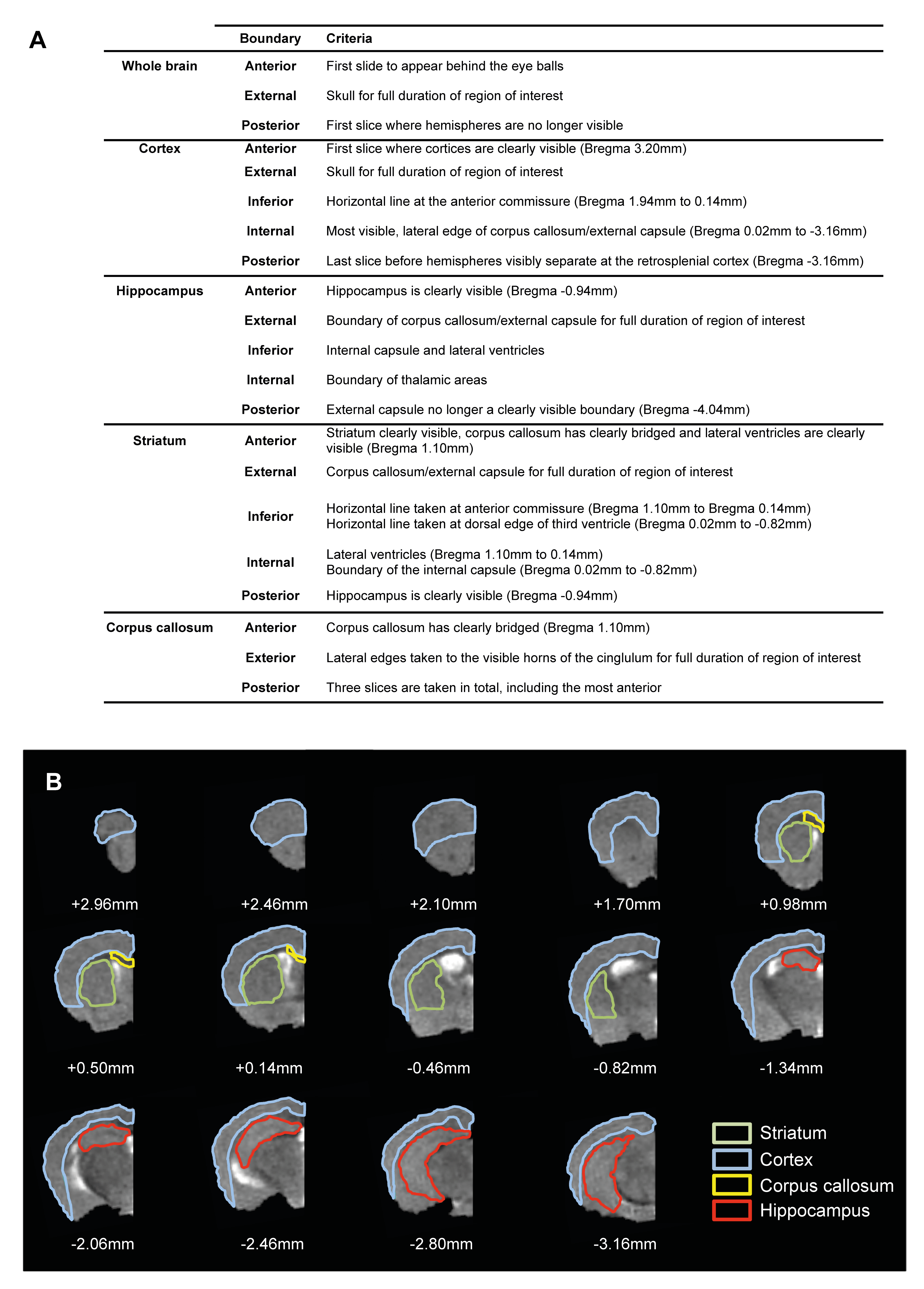

Supplement: Figure S1 — Region of interest delineation criteria used for MRI analysis. (A) Definition of criteria by which regions of interest were delineated onto structural MR images; quoted Bregma reference marks are typically ±0.25 mm as a result of the inconsistency of slice positional matching upon image acquisition. (B) Sample regions of interest delineated onto a 14 week WT mouse brain. (TIF) [file pone.0060012.s001.tif]

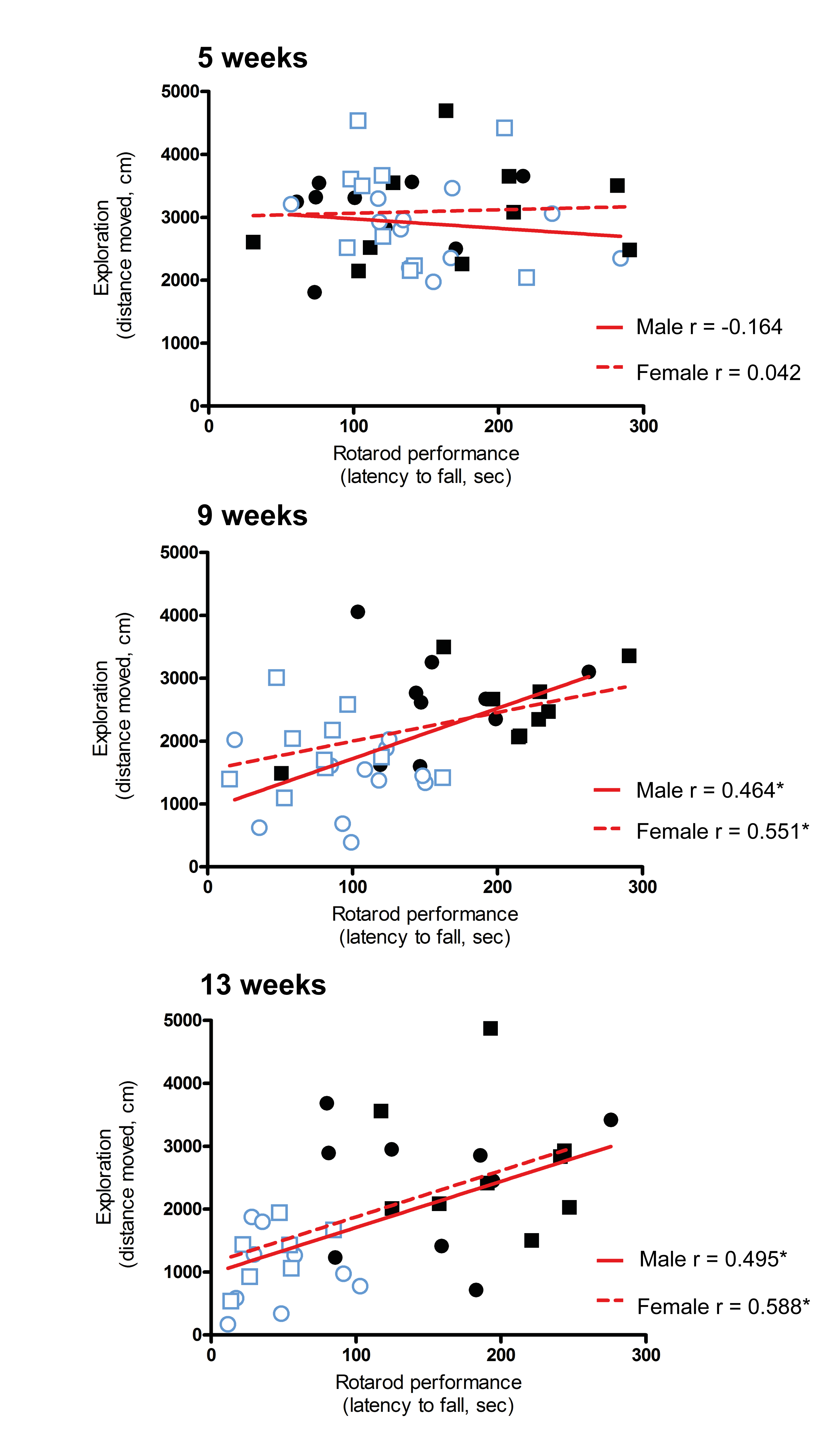

Supplement: Figure S2 — Age related separation of rotarod performance and exploratory activity for WT and R6/2 mice. Latency to fall from a rotarod versus exploration in an open field for both WT and R6/2 at the three behavioral time points, 5, 9 and 13 weeks of age. As the R6/2 s developed age-related deficits at both tasks, the WT and R6/2 data points separated creating significant, positive correlations for both genders when considering all animals together. *p<.05. (TIF) [file pone.0060012.s002.tif]
